# Assessment of Food Safety Knowledge and Practices Among Medical Students

**DOI:** 10.3390/foods14091636

**Published:** 2025-05-06

**Authors:** Maria Nițescu, Mirela Maria Nedelescu, Elena Moroşan, Anca Angela Simionescu, Florentina Ligia Furtunescu, Bianca Eugenia Ştefănescu, Mihail Tusaliu, Eugenia Panaitescu, Alin-Marian Stanciu, Irina Mihaela Stoian

**Affiliations:** 1 Discipline Hygiene and Medical Ecology, Faculty of Medicine, University of Medicine and Pharmacy “Carol Davila”, Dr. Leonte Street, 050463 Bucharest, Romania; maria.nitescu@umfcd.ro (M.N.); irina_mihaela_stoian@umfcd.ro (I.M.S.); 2 Discipline Clinical Laboratory—Hygiene of Nutrition, Faculty of Pharmacy, University of Medicine and Pharmacy “Carol Davila”, Traian Vuia Street, 020956 Bucharest, Romania; 3 Discipline Obstetrics and Gynecology Filantropia, Faculty of Medicine, University of Medicine and Pharmacy “Carol Davila”, 011171 Bucharest, Romania; anca.simionescu@umfcd.ro; 4 Discipline Public Health and Management, Faculty of Medicine, University of Medicine and Pharmacy “Carol Davila”, 050463 Bucharest, Romania; florentina.furtunescu@umfcd.ro; 5 Institute of Life Sciences, University of Agricultural Sciences and Veterinary Medicine, 400372 Cluj-Napoca, Romania; bianca.vodnar@usamvcluj.ro; 6 Department of Food Science and Technology, University of Agricultural Sciences and Veterinary Medicine, 400372 Cluj-Napoca, Romania; 7 ENT Department, Faculty of Medicine, University of Medicine and Pharmacy “Carol Davila”, 020021 Bucharest, Romania; mihail.tusaliu@umfcd.ro; 8 Discipline Medical Informatics and Biostatistics, Faculty of Medicine, University of Medicine and Pharmacy “Carol Davila”, 050474 Bucharest, Romania; eugenia.panaitescu@umfcd.ro; 9 Faculty of Medicine, University of Medicine and Pharmacy “Caro Davila”, 050474 Bucharest, Romania; alin-marian.stanciu@rez.umfcd.ro

**Keywords:** food safety, medical student, microbiological food contamination, foodborne disease, eating behaviors, food choices

## Abstract

Food safety is an important requirement for protecting human health worldwide. In particular, medical students’ education on food safety is essential for them as future physicians, and university education is the first step in acquiring this knowledge. We performed an online survey with 1277 respondents among medical students to assess knowledge, attitudes, and practices (KAPs) related to food safety regarding microbiological contamination. Our findings showed that more than half of the respondents presented a low level of food safety knowledge, with a score between 11–60 points, and only 6% managed to score between 81 and 100 points, which was considered a high level of knowledge. On the contrary, we found that most participants had a high level of good practice: 58% scored more than 25 points, 39% had an average level of good practice (scoring between 21 and 25 points), and 3% of respondents had a low level of good practice (scoring below 21 points). We also noticed a statistically significant difference between total scores of preclinical and clinical years of study among medical students (*p* = 0.005) regarding food safety knowledge. The frequency of cooking was positively correlated with the level of food safety knowledge, but not with food safety practices. Our study shows that better knowledge on food safety is needed among medical students. Improving knowledge and awareness of food safety in relation to microbiological contamination is a good way to protect themselves and to promote the correct food safety knowledge and measures among their patients.

## 1. Introduction

According to a WHO report (2015), about 600 million people worldwide fall ill every year from eating contaminated and/or spoiled food, with 420,000 deaths, including 125,000 deaths in children under 5 years of age in low- and middle-income countries [1]. Foodborne illnesses are caused by both biological agents (bacteria, viruses, parasites, and prions) and chemicals (natural toxins, persistent organic pollutants, and heavy metals). If the symptoms are not serious and the consumer does not end up hospitalized, most cases are often not reported to the relevant authorities.

There are many ways in which food can be contaminated and/or adulterated; it is therefore important that the whole food production chain, from farm to fork, be monitored at critical points to ensure that the food reaching the consumer is safe to eat and does not cause illness or, in some cases, does not cause disease endangering consumers’ lives [2,3,4,5].

In the food supply chain, responsibility for food safety lies with the producer as well as the authorities, who must ensure that the food placed on the market is safe to eat. After buying food, the consumer also plays a role in ensuring food safety by transporting, storing, preparing, and handling food at home. If these activities are not properly managed, they can have a negative impact on food safety and put at risk consumers’ health [2,6].

As future doctors, medical students should acquire sufficient knowledge about food safety. This human health education activity and food safety is an integral part of both pre-clinical and clinical specialties. In accordance with the current curricula for medical students at the Faculty of Medicine of the University of Medicine and Pharmacy “Carol Davila” in Bucharest, they study food and nutritional hygiene in the third year of study. Later, in the higher years of study, in other clinical specialties, medical students have the opportunity to deepen their understanding of nutritional and food hygiene skills.

Concern for efficient communication is an important component of the doctor–patient relationship [7,8]. A doctor’s assessment of a patient starts with an anamnesis, professional and socio-economic conditions afterwards, and then therapeutic interventions and recommendations for healthy living can follow, including information on food safety.

Thus, it is important to assess knowledge and practices among medical students in order to transfer appropriate knowledge to the general population. Recent studies in the field demonstrated that food safety and hygiene education improve the knowledge, attitudes, and practices of students with different specializations, and the necessity of raising awareness among these students about food safety was emphasized [9,10,11]. The necessity of raising awareness among these students about food safety using appropriate interventions was also emphasized [12,13,14].

### 1.1. Hypotheses

Doctors represent a key source of information and education on health among the population. Starting this point, we set out to test the level of knowledge acquired by medical students during undergraduate studies.

In this context, we started our research from 3 hypotheses:

**Hypothesis** **1:** *Medical students have a good level of knowledge regarding food safety (microbiological contamination) as a result of theoretical training during the 6 years of study.*

We expect a good level of knowledge in the study group considering the results of other studies that revealed better knowledge among medical students compared to non-medical profiles [15,16].

**Hypothesis** **2:** *Students in the clinical years of study have a better level of knowledge than those in pre-clinical years of study; students complete the Food and Nutritional Hygiene module and other disciplines related to biological agents that contaminate food by the third year of study.*

**Hypothesis** **3:** *Food safety practices are influenced by the level of knowledge regarding food safety. The importance of education and training translated into good food safety practices was also underlined by other authors [12,17,18,19].*

### 1.2. The Aim of the Study

In the current study, we aimed to assess knowledge and practices regarding food safety in relation to food contamination with biological agents. During this study, we specifically tested their level of knowledge on food safety and the practical application of this knowledge to their personal life, starting with the idea that a future doctor is more likely to convince patients about food safety if he is convinced of the importance of preventive behaviors and already applies them on a personal level.

In addition, we considered this study as a starting point for future adaptation and improvement of the food safety topics covered in the curriculum.

## 2. Materials and Methods

### 2.1. Study Design

We performed an online survey with 1277 respondents among medical students from “Carol Davila” University of Medicine and Pharmacy in Bucharest, Romania, to assess knowledge and practices related to food safety.

### 2.2. Survey Questionnaire

In order to achieve the purpose of this study, a questionnaire with 4 sections was developed to assess different aspects related to food safety, based on models from previously published studies [20,21,22,23,24]. In the first part of the questionnaire, the study was described, and consent was obtained from the respondents for participation in the study, data confidentiality, and personal data processing.

The second section of the questionnaire collected socio-demographic information (age, gender, residence during study, year of study), anthropometric data (weight, height) and information on respondents’ cooking behaviors, type of food purchased, factors influencing food choice, and personal pathological history in relation to food consumption in the recent past (<6 months).

The third section was dedicated to assessing knowledge on food safety. The 10 questions in this section focused on etiologic agents and symptoms of food-borne outbreaks, ways of food contamination, cross-contamination, and measures to prevent contamination. The section contained closed-ended questions with a limited number of answer choices, including 6 single-answer and 4 multiple-choice questions, with 10 marks being awarded for each correct answer. Thus, a student was able to accumulate a total of 100 points from answering the questions in this section.

The fourth section consisted of a set of 10 closed-ended, tri-dichotomous questions: “Yes”, “Every Time”, and “Not Often/No”, by which we collected information about respondents’ food safety practices and habits. The main aspects assessed were food storage and handling, kitchen utensil hygiene, and hand hygiene.

The draft questionnaire was initially tested on a batch of 10 students in order to identify any possible unclear wording of the questions. No questions were deleted or replaced during piloting. Responses from the 10 pilot questionnaires were not included in the final analysis.

The link to the online questionnaire was sent to “Carol Davila” University students through online communication channels (WhatsApp, Facebook, Instagram), and to access the questionnaire, students were asked to log in with their institutional email address, both to confirm their status as a student of the University of Medicine and Pharmacy “Carol Davila” of Bucharest and to restrict the completion to one questionnaire per student. Participation in the study was allowed for students of all years of study, and was voluntary, anonymous (the e-mail address was not collected together with the other information), and without any compensation. Access to the questionnaire was possible for 2 weeks between 1 and 15 November 2021.

The questionnaire was provided to participants in the Romanian language, and the estimated time for completing it was about 5 min.

### 2.3. Statistical Analysis

Data were collected on the Google Forms platform and then exported to an Excel (.xlsx) file. Statistical analysis was performed with SPSS Statistics (version 25, IBM Corp., Armonk, NY, USA).

The analysis performed included descriptive statistics (frequencies, standard deviations, means, medians), comparison of means, and correlations. For all of these, a *p*-value < 0.05 was considered statistically significant, and tests such as ANOVA, the Kolmogorov–Smirnov test, the Kruskal–Wallis test, Cohen’s coefficient calculation, the McNemar test, and standard tests such as the Pearson Chi-square, likelihood, and Student *t*-test were used. In order to identify whether the means of the analyzed parameters were statistically different, the least significant difference (LSD) test was applied.

### 2.4. Ethical Approval

Ethical approval to conduct this study was granted by the Research Ethics Committee of the “Carol Davila” University of Medicine and Pharmacy in Bucharest, Romania.

## 3. Results

A total of 1279 of the medical students accepted the invitation to complete the questionnaire. Subsequently, only 1277 of the questionnaires completed by them were validated, as two respondents did not consent to the processing of their personal data for the purposes of this scientific research. The demographic characteristics of the study group are summarized in Table 1.

A predominance of female respondents was observed, with 979 respondents (76%). Subsequently, it was observed that slightly more than a half of the respondents, 658 (51.5%), fell in the age range of 21–23 years.

The data also revealed that a significant percentage of the respondents lived in a dwelling with a self-contained kitchen where they could prepare their food in a suitable environment.

It was also observed that, in terms of university education stage, the study group was balanced, consisting of 626 medical students (49% of the total respondents) in the preclinical years (years of study I, II, and III) and 651 (51%) of the medical students in the clinical years (years of study IV, V, and VI). The first group of medical students in the preclinical years of undergraduate training (years of study I, II, and III) had not yet completed the module on Food Hygiene, unlike the second group of medical students in the clinical years.

### 3.1. Respondents’ Eating Behaviors and Determinants of Food Choices

**Figure 1:**
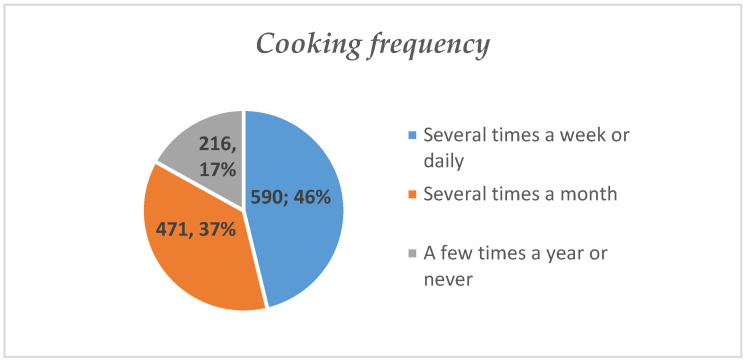
Responses regarding cooking frequency (number of responses; percentage).

The study collected data on certain eating behaviors and practices among medical students (for example, how often they cook their food in a week, month, or year). A total of 590 respondents (46.2%) answered that they cook several times a week or even daily, 471 subjects (36.9%) answered that they cook several times a month, and 216 (16.9% of the total number of respondents) answered that they cook only a few times a year or never (Figure 1).

The frequency of cooking reported by medical students influenced their score within the section related to food safety knowledge, with a high level of significance obtained in the ANOVA test, with a *p*-value = 0.003. Also, applying the LSD tests, we found a statistically significant difference between the respondents who stated that they cook several times a week or daily and the respondents who said they cook several times a year or never (*p* = 0.001). There was also a statistical difference between respondents who reported cooking several times a month and respondents who reported cooking several times a year or never (*p* = 0.006).

Respondents’ food choice and food consumption were mostly influenced by their knowledge and personal preferences, with 1098 respondents (86%) choosing this option. The influence of family was stated to be felt by 626 medical students (49%), and the influence of friends was stated to be felt by 319 (25%) of the respondents. On the other hand, the cost of food influences food choices in the case of 575 respondents (45%); 115 medical students (9%) considered that they are influenced by food marketing in their dietary habits.

The respondents could choose from several response options, and the majority chose at least two answers (i.e., 2 factors) that, in their opinion, influence their food choices and subsequent food consumption.

Following the completion of the questionnaire and data collection, we were able to correlate the gender of respondents and the influence of family on their food choices and subsequent food consumption behavior. Thus, was noted that 54% of the female respondents (529 out of a total of 979 in our survey) are influenced by family members in their food choices and consumption decisions; 117 male respondents out of a total of 293 (40%) said the same.

A correlation could also be made between the age of respondents, the influence of food advertisements on food choices, and subsequent food consumption. A total of 15% of the respondents of an age of between 21 and 23 years said they are most influenced by such food advertisements.

All the respondents aged ˃ 26 years old said that they are not influenced at all by advertisements in their food choices and consumption. About 10% of the respondents aged < 21 years and those aged 24–26 years stated being influenced by these advertisements. It seems that the respondents aged 21–23 years were in the age group most influenced by food advertisements in their food choices and consumption.

Another correlation was obtained between respondents’ gender, the influence of food cost on their choices, and subsequent food consumption. Thus, 176 male respondents (representing 60%) said that they take into consideration the cost of food when buying it, compared to 421 female respondents (43%).

We also investigated respondents’ eating behavior by the preference to purchase packaged or unpackaged food. A total of 106 medical students (8.3%) stated that they prefer to buy unpackaged food, and 432 (33.8%) stated that they prefer pre-packaged foods. More than half of the respondents, 739 (57.9%), considered that food packing is not a criterion when buying food.

A total of 323 female respondents (33%) and 105 male respondents (36%) choose to buy packaged products. Also, 568 female respondents (58%), as well as 167 male respondents (57%), stated that food packaging is not a criterion when buying food.

We also collected data on a possible disease caused by contaminated food in recent months (more precisely, in the previous 6 months): 72 medical students (5.6% of the total respondents) participating in the study answered “yes”. There were also 96 subjects (7.5%) who responded that they “don’t know”/“don’t remember” if they had had an illness caused by contaminated food in the previous 6 months. Thus, 1109 respondents (86.8%) answered that they “did not have” such an illness in the previous 6 months.

Of the reported data, it was found that 80% of the respondents who reported illness due to contaminated food were women, compared with 18% of male respondents. The difference of 2% is due to the only reported case of illness among participants of another gender/non-binary. In regards to the relationship between respondents’ age and recent history (last 6 months), it was found that 100% of respondents aged > 26 years said they had had no episodes of contaminated food-related illness during the previous 6 months. In the age group of 21–23 years, this percent was 6%.

### 3.2. Respondents’ Assessment of Food Safety Knowledge

**Figure 2:**
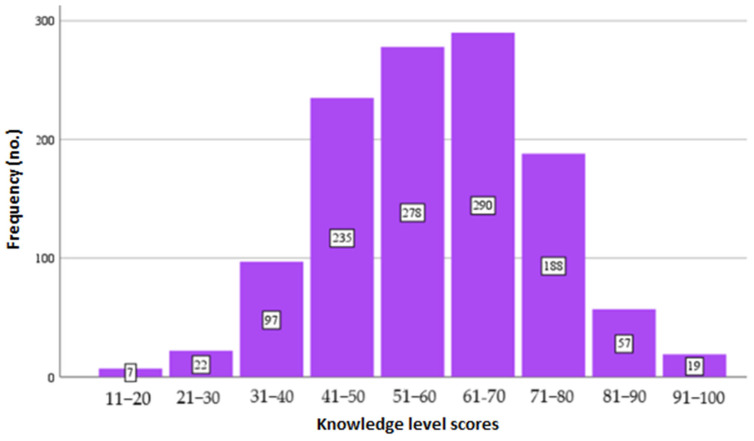
Score obtained in the food safety knowledge test.

The scores obtained by respondents, following the completion of the questionnaire section assessing their knowledge of food safety, had an average value of 58.09 ± 15.021 points; the minimum score obtained was only 11 points, and the maximum score was 100 points (100%) (see Figure 2).

**Figure 3:**
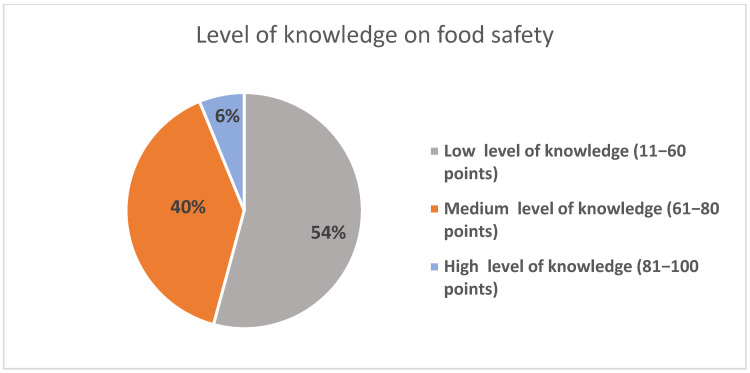
Level of knowledge on food safety among study participants.

Respondents’ scores were grouped into three distinct levels of knowledge—high, medium, and low (Figure 3).

Thus, following the theoretical assessment of food safety, carried out through a set of 10 questions, the following conclusions were reached, establishing that the majority of respondents in the survey had low levels of knowledge. The data are presented in Figure 1 and Figure 2. Only 79 respondents (6%) managed to score between 81 and 100 points; within the survey, this was regarded as a high level of knowledge. In the moderate category, 506 (39%) of the respondents managed to score between 61 and 80 points. More than half of the respondents who participated to the survey fell in the category of a low level of knowledge (with a score of between 11 and 60 points), representing 692 (54%) of them.

Questions that assessed food safety knowledge and the frequency of correct answers are listed in Table 2, following the order of the number of correct answers given by respondents (not in the order in which they appear in the questionnaire).

Thus, according to the collected data, the fewest correct answers were those referring to the identification of pathogens (A—*Salmonella* spp., B—*E. coli*, C—*Clostridium botulinum*, D—*S. aureus*, E—Norovirus), which can be transmitted through contaminated food (only 154 of the respondents answered correctly).

**Figure 4:**
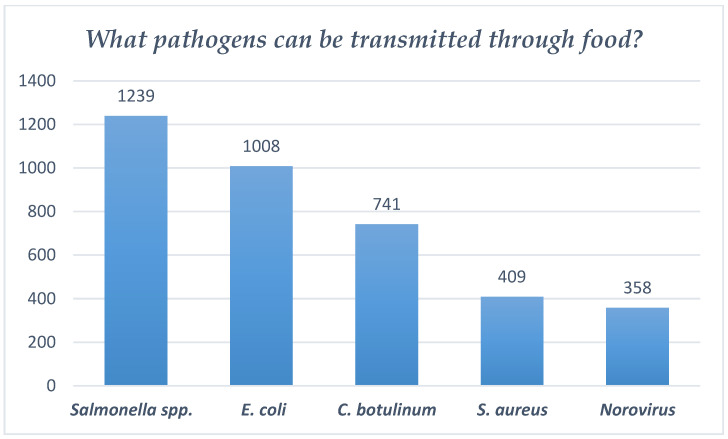
Responses regarding pathogens identified in contaminated food.

In terms of respondents’ knowledge of pathogens identified in contaminated food, it can be seen that *Salmonella* spp. was the pathogen most often identified as transmissible through food, by 1239 respondents (96.9%). Another pathogen, *E. coli*, was correctly identified by 78.9% (1008 respondents), followed by *C. botulinum* (58.3%, 741 respondents). It can also be observed that the pathogens least known by medical students to be transmitted through food were *S. aureus* (409 respondents, 32.3%) and Norovirus (358 respondents, 28%) (see Figure 4).

**Figure 5:**
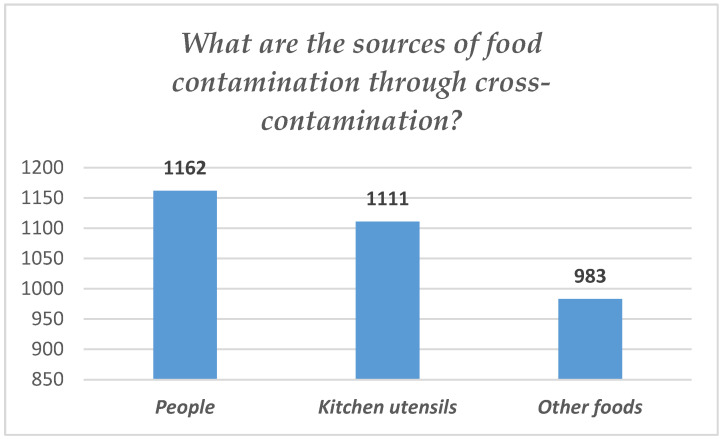
Responses regarding sources of food cross-contamination.

A large proportion of respondents, 1162 (representing 91%), considered humans to be a reliable source of food contamination. Also in a high proportion, 1111 subjects (87%) considered that during the process of cross-contamination of food, possible pathogens present on kitchen utensils could come into contact with food. A total of 77% of medical students (983) considered that there is a risk of cross-contamination from one food to another (see Figure 5).

More than half of the respondents, 691 medical students (54.1%), answered correctly about the length of time to keep food in the refrigerator (3–4 days). The overestimation of food perishability was found in the responses of 518 respondents (40.6%), who considered that the optimal amount of time that food can be kept in the refrigerator is shorter, only 1–2 days. In addition, 62 respondents (4.9%) underestimated the perishability of food and considered that food can be kept in the refrigerator for a longer period of time (5–6 days). A small proportion of the respondents, six subjects (0.5%), considered that food can be kept in the refrigerator for more than 6 days.

Nearly half of the respondents, 577 subjects (45.2%), answered correctly on how long food can be kept out of the refrigerator after thermal processing (1–2 h). Overestimation of storage outside the refrigerator at 3–4 h was found in the responses of 551 medical students (43.2%). Also, 119 respondents (9.3%) considered that food can be stored outside the refrigerator for 5–6 h after thermal processing, and 30 subjects (2.4%) considered that the food could be kept out of the refrigerator for more than 6 h.

Furthermore, we investigated the knowledge about proper defrosting of food. A very small number of respondents—13 subjects (1%)—provided the wrong answer that using a microwave oven is an option for defrosting food correctly. A traditional option for defrosting, which involves placing food in a bowl of water at room temperature (setting up an environment favoring the development of pathogens) was chosen by 306 subjects (24%). Other respondents, 396 (31%), thought that the correct way to defrost food is to keep it at room temperature (i.e., at a temperature of 21–22 °C). Less than half of the respondents (549, representing 43% of the total respondents) chose the correct option, that “food defrosts in the refrigerator at 0–4 °C”.

When asked whether refrigeration destroys all pathogens that can be found in food, 856 respondents (67%) chose the correct answer. The remaining respondents ticked one of the wrong answers: 383 (30%) chose the answer “only destroys pathogens that are vulnerable to low temperatures”, 26 (2%) chose the answer “no, on the contrary, it facilitates the multiplication of pathogens”, and 13 (1%) chose the answer “yes, it destroys all pathogens”.

Only 472 medical students participating in the survey (37%) believed that “you can’t rely on smell, look, or taste to identify a food possibly contaminated with bacterial toxins”. A third of the total number of medical students, 434 respondents (34%), thought that smell helps to identify food possibly contaminated with bacterial toxins. Nearly a quarter of respondents, 294 (23%), felt that only by the sensorial characteristics can a food possibly contaminated with bacterial toxins be identified.

A small percentage, 5% (64 respondents), considered that “to identify a possibly contaminated food, I would have to taste it”—of course, this answer is wrong and is certainly not recommended if we suspect bacterial toxin contamination of a particular food.

Out of the total number of medical students participating in the study, 511 subjects (40%) thought that “foods with high water content favor the multiplication of bacteria”, taking into account the specific environment provided by food. Over half, 677 respondents (53%), thought that products with an increased sugar content would favor bacterial multiplication. Indeed, sugars can act as a substrate for bacteria, but their increased concentration creates a hypertonic environment that affects microorganisms, preventing them from multiplying and thriving.

A practical example of using increased sugar concentrations to prevent bacteria multiplication is preparing jams and jellies. Similarly, another form of food preservation is the use of high salt concentrations—for example, in the preparation and preservation of pickles. To a small extent, about 3% of respondents (38 subjects) thought that foods with high salt concentrations favor bacteria multiplication. Also, a small percentage (5%, 64 respondents) thought that products with low water content would encourage bacteria to multiply.

As regards the most common symptoms of food poisoning **(A—nausea, C—vomiting, D—diarrhea, E—fever, F—abdominal pain)**, the most easily identifiable for respondents were vomiting (98%), diarrhea (97%), nausea (95%), and abdominal pain (94%).

However, there were also some symptoms that made the medical students participating in the study feel uncomfortable. Thus, 1006 (78.8% of the total respondents) considered fever a symptom in the clinical picture of microbial food poisoning; another 230 respondents (18%) added constipation to this clinical picture, along with paresthesia, which was the choice of 102 respondents (8%).

In terms of measures that can be used to avoid food contamination (**A—Personal hygiene**, **B—Environmental hygiene**, **C—Separation of thermally prepared food from non-thermally prepared food**, **D—Separation of vegetables from meat**, **E—Refrigeration of food**, **F—Thermal preparation of food**), 792 medical students (62%) felt that separating vegetables from meat was the right way to avoid food contamination. In contrast, medical students did not consider other measures to avoid food contamination, such as food refrigeration (67%) and the separation of heat-processed and non-heat-processed food (69%).

The majority of medical students considered personal hygiene (1162 respondents, representing 91%) to be “the most appropriate method to avoid contamination”, followed by environmental hygiene (1124, 88%) and thermal preparation of food (1098, 86%).

### 3.3. Results of the Analysis on Possible Correlations Between Participants’ Knowledge Level, Socio-Demographic Characteristics, and Eating Behavior

The gender of participants did not influence the level of food safety knowledge (*p* = 0.704). It was observed that females had an average score of 59.3 ± 15.0, males scored 58.4 ± 14.9 points, and those who declared another sex scored 58.4 ± 23.2 points (see Table 3).

Taking into account the medical educational stage of respondents’ pre-clinical or clinical undergraduate training, a statistically significant difference was noticed between the total scores of these two sub-groups of medical students (*p* = 0.005) (Table 3).

We found statistically relevant differences for some questions between the two sub-groups of medical students depending on the year of study (see Table 4). These data were highlighted using Pearson Chi-square and likelihood tests. A statistically significant difference of 3.6% was observed in favor of respondents in the clinical years of study regarding correctly identifying all possible food pathogens (*p* = 0.04). This trend of a slightly higher percentage in the case of students from the clinical years compared to the pre-clinical years was also maintained for the questions on correctly defrosting food (8% higher), correctly identifying the most common symptoms of contaminated food poisoning (19%), and correctly identifying all methods to avoid food contamination (10.8%). The only statistically relevant difference in favor of students from the preclinical years of study, who answered correctly at a higher percentage than those in the clinical years of study, was for the question regarding food that may favor the multiplication of bacteria, with a percentage difference of 6.1%. (*p* = 0.026).

### 3.4. Respondents’ Assessment of Food Safety Practices

The food safety practices assessment was conducted through a set of 10 questions, for which the respondents were able to obtain a maximum score of 30 points. In our study group, the mean score obtained was 25.8 ± 2.4 points, the minimum score was 15 points, and the highest score was 27 points.

The scores obtained by the study participants were grouped into three distinct levels of good practice: high, medium, and low. Thus, following the assessment of good practices regarding food safety, it was found that the majority of participants had a high level of good practices in terms of food safety. A total of 741 participants (58%) scored more than 25 points, 498 participants (39%) had an average level of good practices (scoring between 21–25 points), and the remaining 38 participants (3%) had a low level of good practices (scoring below 21 points).

The least respected food safety practices were the following, according to the questionnaire responses completed by the medical students:

- “*Not to consume thermally prepared foods that have been at room temperature for more than 2 h*”, which only a small proportion of respondents, 191 students (15%), respected;
- “*Not washing eggs until their use*”, reported by 532 respondents (41%).

On the other hand, the most respected practices were:

- “*Washing fruits and vegetables before eating*”, reported by 1230 respondents (96%);
- “*Washing hands with soap and water before preparing food*”, a response given by 1210 respondents (95%).

The data are summarized in Table 5.

Applying the ANOVA test, a statistical difference (*p* = 0.009) was found between males and females when comparing mean scores of food safety practices by gender.

In terms of differences between male and female respondents to certain questions (the data are summarized in Table 5), it was found that female respondents generally have a higher level of good practices than male respondents regarding washing hands with soap and water before preparing food (96% of female respondents do this every time compared to 90% of male respondents; *p*-value = 0.0008), washing hands with soap and water after preparing food (83% of female respondents do this every time compared to 77% of male respondents; *p*-value = 0.025), and washing the knife and mincer after each cut, especially after raw meat preparation (70% of female respondents do this every time compared to 61% of male respondents; *p*-value = 0.009).

A higher level of good practices was identified among men only in relation to wearing accessories (rings and bracelets) during food preparation (57% of female respondents never wear these accessories compared to 82% of male respondents; *p*-value < 0.001). However, this finding is not relevant, as it is known that women wear this jewelry more often (the data are summarized in Table 6).

The frequency of cooking did not influence the score of food safety practices. Thus, respondents who stated that they cook a few times a week or daily had an average score of 25.9 ± 2.4, respondents who reported cooking several times a month had an average score of 25.8 ± 2.5, and those who cooked several times a month or never had an average score of 25.6 ± 2.5, with no statistically differences (*p* = 0.212 was obtained applying ANOVA test).

The average score obtained by the pre-clinical students in the section regarding food safety practices was 25.8 ± 2.5 points, and the average score obtained by the medical students in the clinical years of study was 25.8 ± 2.3 points, with no statistically significant differences in Student’s *t*-test in terms of the university education level of medical students participating in the study (*p* = 0.666).

We found a statistically relevant difference between the level of good practice of medical students participating in the study and the recent history of food poisoning (*p* = 0.002). The average score of those who had, in the previous 6 months, an illness caused by contaminated food was 24.9 ± 2.9 points, lower than the comparison group, which had an average score of 25.8 ± 2.4 points.

## 4. Discussion

In order to assess the knowledge and practices concerning key food safety knowledge and measures among medical students, we used a KAP-type questionnaire elaborated for the scope of this study. These questionnaires are widely used in different fields, such as evaluating food safety among food handlers or consumers [25,26,27,28,29,30,31,32] and among hospital personnel [19,33,34], assessing nutrition and dietary habits in different categories of the population [35,36,37,38], and investigating other medical and non-medical areas [39,40,41,42,43].

**More than half (54%) of the medical students had a low level of food safety knowledge in the study group. Thus, Hypothesis no. 1 was not confirmed**. We consider that this low level of knowledge is due to the fact that half of the study participants had not accomplished the Hygiene module and did not fully understand the concepts of food safety. Only 79 respondents (6.15%) scored more than 81 points out of total of 100 points. These findings are in accordance with the results of previous studies conducted in other countries [16,44,45]. Ma et al. reported similar results among college students in China. Even if the knowledge level was low, the attitude towards food safety was positive, and students’ practice was moderate and acceptable [45]. Also, a study conducted in Spain found an adequate knowledge among Health Sciences students of how to keep previously cooked meals in order to prevent foodborne illness among consumers [24]. In contrast, our study revealed that the students had a good level of knowledge regarding the ways of cross-contamination, the methods used to avoid food contamination, and the most common symptoms of foodborne diseases.

Among the determinants that influence food safety responses are age, gender, education level, and study specialty [21,45]. Gender did not influence the level of knowledge in our study group. This finding is not in line with other studies—for example, women seem to be aware of the risk of cross-contamination, while men have better knowledge of storage temperature and food preservation [24].

Analyzing the scores obtained by study participants in the section regarding food safety knowledge revealed a statistically significant difference between total scores of medical students from the pre-clinical stage and the clinical stage. **Hypothesis no. 2 was confirmed, with our results demonstrating that students in the clinical years of study had a better level of knowledge than those in the pre-clinical years of study.**

In terms of food safety practices, medical students’ responses did not vary greatly between clinical and preclinical years of university education. The correlation between the degree of education and the level of food safety knowledge and practice was also investigated by other authors. Luo et al. found inadequate knowledge and inappropriate behavior and practices among medical students and nursing students. It seems that the nursing students had lower scores in the knowledge section but higher scores for attitude and practices regarding food safety issues compared to medical students. However, students were more concerned about the presence in food of pesticide residues, veterinary drugs, or the transfer of plastics from the materials in contact with food [16].

**The level of food safety practices in the studied group was high.** More than half of the respondents, 766 medical students (58.5%), scored > 25 points out of a total of 30 possible points on the questionnaire. However, there was no statistically relevant difference between female and male respondents.

The frequency at which the respondents cook, according to the collected answers, did not influence the level of good practices. Our survey data identified that people who cook a few times a week or more often had an average score of 59.96 points, slightly higher than the people who cook a few times a year or never, with an average score of 55.99 points, but no statistically significant differences were found.

**Hypothesis no. 3 was not confirmed**. Applying the Pearson correlation test, **we did not identify a correlation between the level of knowledge and the level of good practices** (*p* = 0.054, slightly above the 0.05 threshold).

**Figure 6:**
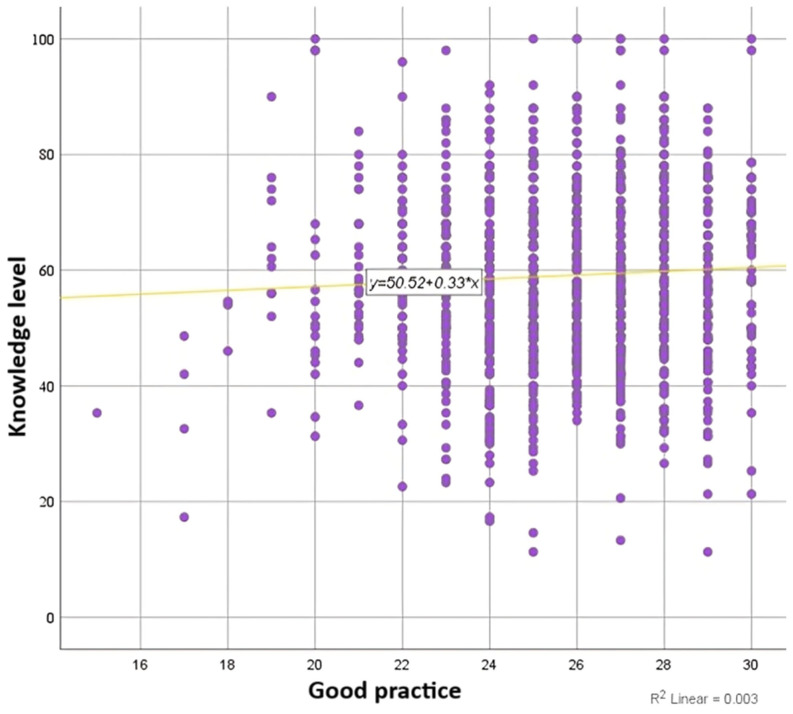
Correlation between level of knowledge and level of good food safety practices among the medical students participating in the study.

Furthermore, statistically relevant correlations (*p* < 0.005) were found using the McNemar test by comparing respondents’ knowledge with practices (Figure 6).

For a better description of the correlation between the level of knowledge and good food safety practices, we collected and compared information through two different questions addressed to the study participants: a question revealing the knowledge of the participants, and another assessing practice concerning a given situation.

Thus, the appropriate answers to the knowledge test and the percentages of adequate answers to the good practices test are presented in parallel below:

- Regarding the risk of E. coli infection through consumption of unwashed fruit and vegetables, 1009 respondents (79%) are aware of the possibility of the presence of pathogen agents on the food. The vast majority of respondents, 1226 (96%), confirmed that they wash fruit and vegetables every time before eating.
- However, as regards the risk of Salmonella infection, the vast majority of respondents, 1239 subjects (97%), are aware of the possibility of Salmonella on food; however, only about one third of the respondents, 383 subjects (30%), wash eggshells each time before using them in food preparation. Salmonella represents the most common cause of foodborne outbreaks in the European Union [46,47,48], and the most frequently contaminated food products in the region are egg products [49].
- Data collected about cross-contamination, caused by the person cooking the food touching more than one surface and the food with their own hands, showed that the majority of respondents, 1213 (95%), wash their hands every time before starting to prepare food. Also, a significant number of medical students participating in the study, 1137 subjects (89%), wash their hands every time after handling raw meat (pork, chicken, poultry), and a significant number of respondents, 1047 (82%), wash their hands every time after food preparation. Also, 230 respondents (18%) do not wash their hands after cooking. Of all respondents, 830 (65%) indicated being aware of cross-contamination danger.
- About half of the survey respondents, 690 subjects (54%), indicated knowing the optimal storage range for perishable foods in the refrigerator. However, 1137 respondents (89%) answered that they read the “use by” date on the food labels and respect it.
- Less than half of the participants, 575 medical students (45% of the total number of respondents), stated that they know the maximum time to keep thermally prepared food out of the refrigerator at room temperature to avoid contamination. Out of the total number of respondents, only 192 subjects (15%) respect the optimal time (2 h) of keeping thermally prepared food outside the refrigerator; the remaining respondents—1085 (85%)—do not respect the optimal time and eat the food even after it has been left for longer at room temperature (more than 2 h). In terms of food storage and the benefits of storing food in a refrigerator, 856 students (67%) answered correctly and knew that pathogens are not inactivated/eliminated by storing food in the refrigerator and that this can encourage stagnation or their multiplication in food. A total of 677 respondents (53%) answered that they read the information on food packaging about storage conditions to properly manage food and refrigeration.
- Of the total number of respondents, 55% of them (702 students) seemed to be aware of all prevention methods of food contamination. However, only 792 students (62%) remove their accessories (rings and bracelets) before food preparation begins. Thus, a significant number of respondents, 472 (37%), are exposed to the risk of food contamination through accessories worn during the cooking process. Jewelry hides dirt and bacteria, and can represent a risk of physical and chemical hazards. For this reason, food handlers should not wear rings, earrings, or watches [50].

Good practice in terms of food safety is important, as is recognizing the significance of knowledge and attitudes related to food safety [51].The assessment of food hygiene knowledge and practices is most often carried out among food industry personnel, including those working in restaurants, in order to prevent and control the occurrence of foodborne diseases among consumers [26,27,28,29,52,53]. Food business operators are responsible for ensuring that food complies with the relevant microbiological criteria set by legislation [54]. During all stages of production, processing, storage, and distribution of food, food business operators must take measures in accordance with the HACCP (Hazard Analysis and Critical Control Point) principles and maintain good hygiene practices. They must therefore ensure that the delivery, handling, and processing of raw materials and food are carried out in such a way that the hygiene criteria of the process are met [54].

However, in our country, the highest occurrence of foodborne outbreaks is caused by food prepared at home, not food prepared in restaurants, and the most frequent foodborne outbreaks are represented by family outbreaks [55,56]. Contributing factors are food preparation practices, behaviors, and environmental conditions that lead to pathogens getting into food, growing in food, or surviving in food. Also, conservation and cooking methods, as well as food physico-chemical properties, are important parameters for determining the safety of food products [57,58].

Our study has certain limitations related to the selection of the study group, which was voluntary rather than randomized recruitment, and the fact that there is a net predominance of female subjects, because in our university women predominate over men. In addition, our university Hygiene curriculum includes only a 2 h course on food safety, and some aspects of food microbiological contamination are presented in only four practical works, which differ from the curricula of other Romanian medical universities. For these reasons, we cannot extend the results to the national level. To better understand the relationship between knowledge and practices, it was also useful to investigate attitudes towards food safety. To achieve a better response rate among the study group, we chose to assess only food safety knowledge and practices, leaving the way open for future research to clarify these aspects. It is also crucial to reassess knowledge and practices related to microbiological contamination of food and preventive measures after graduating from medical school.

## 5. Conclusions

A good level of food safety knowledge within the university stages of medical students is a necessity in all kinds of medical specialties in order to transfer proper knowledge to patients.

Medical students are a unique and very important audience for education on food safety and, subsequently, for the prevention of foodborne diseases, in terms of both patient education and the protection of human health.

Even though the respondents’ knowledge on food safety was rather limited (a low level of knowledge), students reported high level of food safety practice.

In addition, we consider this study a good starting point for future change to improve the food safety topics covered by the university curricula in order to achieve better knowledge and attitudes about food safety.

The results highlighted the practical need to allocate more hours to food safety education and training in medical students’ courses, as well as the need to raise awareness of the importance of food safety in their future profession.

## Figures and Tables

**Table 1:** Characteristics of respondents included in the study (No. = 1277).

Characteristics of Respondents	Number (%)
**Gender**	
Women	979 (76.7%)
Men	293 (22.9%)
Other gender	5 (0.4%)
**Age**	
Age < 21 years	658 (51.5%)
Age 21–23 years	380 (29.8%)
Age 24–26	224 (17.5%)
Age ˃ 26 years	15 (1.2%)
**Living space**	
Permanent residence	497 (38.9%)
Property rented during university studies	472 (37%)
Room at the student dormitory of the University of Medicine and Pharmacy “Carol Davila” Bucharest	253 (19.8%)
Private dormitory	55 (4.3%)
**Year of study**	
**Preclinical university studies:**	**626 (49%)**
Year I	89 (7%)
Year II	294 (23%)
Year III	243 (19%)
**Clinical university studies:**	**651 (51%)**
Year IV	178 (14%)
Year V	243 (19%)
Year VI	230 (18%)

**Table 2:** The frequency of correct answers from the food safety knowledge section (No. = 1277).

Question	Frequency of Correct Answers (No.)
Q1. Which of the following pathogens can be transmitted through food?	154
Q7. How can you tell if your food is contaminated with bacterial toxins?	473
Q8. What makes bacteria multiply?	511
Q5. How do you defrost a food product correctly?	556
Q4. How long should we keep food outside the refrigerator after it has been thermally processed?	577
Q3. What is the optimal storage interval for perishable foods in the refrigerator?	691
Q10. What methods should be used to avoid food contamination?	709
Q9. What are the most common symptoms of food poisoning?	711
Q2. Cross-contamination refers to the process of transfer pathogen agents from other sources in food?	835
Q6. Does refrigeration eliminate all pathogens that may be present in food?	862

**Table 3:** Comparison of food safety knowledge by gender and year of study.

	Frequency (no.)	Average Score	SD	Minimum Score	Maximum Score	*p*-Value
**Gender**						
Women	979	59.3	15.0	11	100	
Male	293	58.4	14.9	11	100	0.704
Other	5	58.4	23.2	17	72	
**Year of study**						
Preclinical ^1^	626	57.9	15.0	11	100	0.005 *
Clinic ^2^	651	60.2	15.0	14	100	

^1^ The preclinical years of study comprise years I, II, and III of undergraduate study. ^2^ The clinical years of study comprise years IV, V, and VI of undergraduate study. * *p* < 0.05 with statistical significance.

**Table 4:** Correct answers related to food safety knowledge by the stage of year of study (No. = 1277).

Question	Preclinical Years		Clinical Years		
	Frequency (N = 626)	Percentage (%)	Frequency (N = 651)	Percentage (%)	*p*-Value
Q1.Which of the following pathogen agents can be transmitted through food?	64	10.2%	90	13.8%	0.048 *
Q2. Cross-contamination refers to the process of transfer of pathogens from other food sources?	408	65.2%	427	65.6%	0.876
Q3. What is the optimal storage interval for perishable foods in the fridge?	344	55%	347	53.3%	0.554
Q4. How long should we keep food outside the refrigerator after it has been thermally processed?	285	45.5%	292	44.9%	0.809
Q5. How do you defrost a food product correctly?	247	39.5%	309	47.5%	0.004 *
Q6. Does refrigeration eliminate all pathogens that may be present in food?	421	67.3%	441	67.7%	0.852
Q7. How can you tell if your food is contaminated with bacterial toxins?	237	37.9%	236	36.3%	0.552
Q8. What promotes breeding bacteria?	270	43.1%	241	37%	0.026 *
Q9. What are the most common symptoms of food poisoning?	288	46%	423	65%	<0.0001 *

* *p* < 0.05 with statistical significance.

**Table 5:** Section on the food safety practices assessment.

Question	Every Time		Sometimes		No	
	Frequency (No.)	Percentage (%)	Frequency (No.)	Percentage (%)	Frequency (No.)	Percentage (%)
Q1. Do you wash your hands with soap and water before preparing food?	1210	94.8%	66	5.2%	1	0.05%
Q2. Do you wash your hands with soap and water after handling raw meat (pork, chicken, seafood)?	1143	89.5%	119	9.3%	15	1.2%
Q3. Do you wash your hands with soap and water after food preparation?	1047	82%	206	16.1%	24	1.9%
Q4. Do you eat food that has been left at room temperature for more than 2 h?	420	32.9%	666	52.1%	191	15%
Q5. Do you wash fruits and vegetables before eating them?	1230	96.3%	45	3.5%	2	0.12%
Q6. Do you wear accessories such as rings or bracelets while cooking?	134	10.5%	345	27%	798	62.5%
Q7. Do you wash the knife and cutting board after each use, especially after cutting meat?	865	67.7%	328	25.7%	84	6.6%
Q8. Do you wash eggs before using them for meal preparation?	383	30%	362	29%	532	41%
Q9. Do you read and observe the storage conditions on food product packaging?	686	53.8%	563	44.1%	28	2.2%
Q10. Do you read and respect the expiration date on food product labels?	1145	89.7%	125	9.8%	7	0.6%

**Table 6:** Comparison of level of food safety practices by gender (No. = 1277).

Question	Women		Male		Other		
	Frequency (No. = 979)	Percentage (%)	Frequency No. = 293)	Percentage (%)	Frequency (No. = 5)	Percentage (%)	*p*-Value
Q1. Do you wash your hands with soap and water before preparing food?	941	96.1%	265	90.4%	4	80%	0.0008 *
Q2. Do you wash your hands with soap and water after handling raw meat (pork, chicken, seafood)	880	89.9%	259	88.4%	4	80%	0.6318
Q3. Do you wash your hands with soap and water after preparing food?	815	83.6%	226	77.1%	3	60%	0.025 *
Q4. Do you eat food that has been left at room temperature for more than 2 h?	154	15.7%	36	12.3%	0	0%	0.1843
Q5. Do you wash fruits and vegetables before eating them?	955	97.5%	271	92.5%	5	100%	0.0003 *
Q6. Do you wear accessories such as rings or bracelets while cooking?	555	56.7%	241	82.3%	2	40%	<0.0001 *
Q7. Do you wash the knife and cutting board after each use, especially after cutting meat?	684	69.9%	179	61.1%	2	40%	0.009 *
Q8. Do you wash eggs before using them for meal preparation?	296	30.2%	86	29.4%	1	20%	0.8428
Q9. Do you read and observe the storage conditions on food product packaging?	512	52.3%	171	58.4%	3	60%	0.1693
Q10. Do you read and respect the expiration date on food product labels?	881	90%	260	88.7%	4	80%	0.6751

* *p* < 0.05 with statistical significance.

## Data Availability

The original contributions presented in the study are included in the article; further inquiries can be directed to the corresponding authors.

## References

[1] World Health Organization (2024). Food Safety Factsheet. https://www.who.int/news-room/fact-sheets/detail/food-safety.

[2] Fung F., Wang H.-S., Menon S. (2018). Food safety in the 21st century. Biomed. J..

[3] European Commission (2020). A Farm to Fork Strategy for a Fair, Healthy and Environmentally Friendly Food System. https://food.ec.europa.eu/horizontal-topics/farm-fork-strategy_en.

[4] FAO, IFAD, UNICEF, WFP, WHO (2020). The State of Food Security and Nutrition in the World 2020. Transforming Food Systems for Affordable Healthy Diets.

[5] Huang Y., Wang X., Rui Wang R., Min J. (2022). Analysis and Recognition of Food Safety Problems in Online Ordering Based on Reviews Text Mining. Wirel. Commun. Mob. Comput..

[6] Chen L., Guttieres D., Levi R., Paulson E., Perakis G., Renegar N., Springs S. (2021). Public health risks arising from food supply chains: Challenges and opportunities. Nav. Res. Logist..

[7] Forsey J., Ng S., Rowland P., Freeman R., Li C., Woods N.N. (2021). The Basic Science of Patient-Physician Communication: A Critical Scoping Review. Acad. Med..

[8] Zhang X., Li L., Zhang Q., Le L.H., Wu Y. (2024). Physician Empathy in Doctor-Patient Communication: A Systematic Review. Health Commun..

[9] Alghafari W.T., Arfaoui L. (2022). Food safety and hygiene education improves the knowledge, attitudes, and practices of Saudi dietetics students. Bioinformation.

[10] Keczeli V., Kóró M., Tóth V., Csákvári T., Tisza B.B., Szántóri P., Asztalos Á.C., Verzár Z., Kisbenedek A.G. (2024). Food Safety and Food Hygiene Knowledge of Hungarian University Students. Int. J. Environ. Res. Public Health.

[11] Vuksanović N., Demirović Bajrami D., Petrović M.D., Jotanović Raletić S., Radivojević G. (2022). Knowledge About Food Safety and Handling Practices—Lessons from the Serbian Public Universities. Zdr. Varst..

[12] Fahmy E., Ragab H., Abd el-Sattar E. (2024). Food Safety Knowledge and Handling Practice among Medical Students at Zagazig University. Zagazig Univ. Med. J..

[13] Liu J., Wang S., Wang Z., Chen S. (2024). Research on online public opinion dissemination and emergency countermeasures of food safety in universities-take the rat head and duck neck incident in China as an example. Front. Public Health.

[14] Cai Z., Luo X., Xu X., Shi Z., Reis C., Sharma M., Hou X., Zhao Y. (2023). Effect of WeChat-based intervention on food safety knowledge, attitudes and practices among university students in Chongqing, China: A quasi-experimental study. J. Health Popul. Nutr..

[15] Halwani M. (2023). A Study to Assess Basic Food Safety Knowledge among University Students. Food Nutr. Sci..

[16] Luo X., Xu X., Chen H., Bai R., Zhang Y., Hou X., Zhang F., Zhang Y., Sharma M., Zeng H. (2019). Food safety related knowledge, attitudes, and practices (KAP) among the students from nursing, education and medical college in Chongqing, China. Food Control.

[17] Putri M.S., Susanna D. (2021). Food safety knowledge, attitudes, and practices of food handlers at kitchen premises in the Port ’X’ area, North Jakarta, Indonesia 2018. Ital. J. Food Saf..

[18] Marklinder I., Wersén V., James K. (2025). Food safety and healthcare professionals: The need for education and research. Food Control.

[19] Guennouni M., Admou B., Bourrhouat A., El Khoudri N., Zkhiri W., Talha I., Hazime R., Hilali A. (2022). Knowledge and Practices of Food Safety among Health Care Professionals and Handlers Working in the Kitchen of a Moroccan University Hospital. J. Food Prot..

[20] Stratev D., Odeyemi O.A., Pavlov A., Kyuchukova R., Fatehi F., Bamidele F.A. (2017). Food safety knowledge and hygiene practices among veterinary medicine students at Trakia University, Bulgaria. J. Infect. Public Health.

[21] Azanaw J., Dagne H., Andualem Z., Adane T. (2021). Food Safety Knowledge, Attitude, and Practice of College Students, Ethiopia, 2019: A Cross-Sectional Study. BioMed Res. Int..

[22] Türkistanl T.T., Sevgili C. (2018). Food hygiene knowledge and awareness among undergraduate maritime students. Int. Marit. Health.

[23] Islam M.N., Hassan H.F., Amin M.B., Madilo F.K., Rahman M.A., Haque M.R., Aktarujjaman M., Farjana N., Roy N. (2022). Food safety and handling knowledge and practices among university students of Bangladesh: A cross-sectional study. Heliyon.

[24] Garayoa R., Córdoba M., García-Jalón I., Sanchez-Villegas A., Vitas A.I. (2005). Relationship between consumer food safety knowledge and reported behavior among students from health sciences in one region of Spain. J. Food Prot..

[25] Da Vitória A.G., de Souza Couto Oliveira J., de Almeida Pereira L.C., de Faria C.P., de São José J.F.B. (2021). Food safety knowledge, attitudes and practices of food handlers: A cross-sectional study in school kitchens in Espírito Santo, Brazil. BMC Public Health.

[26] Al-Wehedy A., Faisal S., Omar N., Elsherbeny E. (2021). Knowledge, attitude and practice of food handlers towards food safety. Egypt. J. Occup. Med..

[27] Begum M., Alam M.J., Parikh P., De Steur H. (2025). Understanding food safety knowledge, attitude, and practices of consumers and vendors: An umbrella review. Food Control.

[28] Halim-Lim S.A., Mohamed K., Sukki F.M., David W., Ungku Zainal Abidin U.F., Jamaludin A.A. (2023). Food Safety Knowledge, Attitude, and Practices of Food Handlers in Restaurants in Malé, Maldives. Sustainability.

[29] Zanin L.M., da Cunha D.T., de Rosso V.V., Capriles V.D., Stedefeldt E. (2017). Knowledge, attitudes and practices of food handlers in food safety: An integrative review. Food Res. Int..

[30] Alzhrani W.F., Shatwan I.M. (2024). Food Safety Knowledge, Attitude, and Practices of Restaurant Food Handlers in Jeddah City, Saudi Arabia. Foods.

[31] Kunadu A.P., Ofosu D.B., Aboagye E., Tano-Debrah K. (2016). Food safety knowledge, attitudes and self-reported practices of food handlers in institutional foodservice in Accra, Ghana. Food Control.

[32] Medeiros L.C., Hillers V.N., Chen G., Bergmann V., Kendall P., Schroeder M. (2004). Design and development of food safety knowledge and attitude scales for consumer food safety education. J. Am. Diet Assoc..

[33] Juarez-Medel C.A., Toledo-Ortiz R., Gonzalez-Rojas J.M., Reyna-Alvarez M.A., Olivares-Trejo M.P., Arriaga-Rodriguez S., Alvarado-Castro V.M., Esteves-Garcia F., Davalos-Martinez A., Diego-Galeana A.J.I. (2024). Primary healthcare knowledge, attitudes, and practices among the personnel of a secondary hospital in Acapulco, Mexico. Clin. Epidemiol. Glob. Health.

[34] Shacho E., Ambelu A., Yilma D. (2024). Knowledge, attitude, and practice of healthcare workers towards healthcare- associated infections in Jimma University Medical Center, southwestern Ethiopia: Using structural equation model. BMC Health Serv. Res..

[35] Fu L., Shi Y., Li S., Jiang K., Zhang L., Wen Y., Shi Z., Zhao Y. (2024). Healthy Diet-Related Knowledge, Attitude, and Practice (KAP) and Related Socio-Demographic Characteristics among Middle-Aged and Older Adults: A Cross-Sectional Survey in Southwest China. Nutrients.

[36] Hammouh F., Abdullah M., Al-Bakheit A., Al-Awwad N.J., Dabbour I., Al-Jawaldeh A. (2023). Nutrition Knowledge, Attitudes, and Practices (KAPs) among Jordanian Elderly-A Cross-Sectional Study. Nutrients.

[37] Angarita-Díaz M.D.P., Colmenares-Pedraza J.A., Agudelo-Sanchez V., Mora-Quila J.A., Rincón-Mejia L.S. (2024). Knowledge, Attitudes, and Practices Associated with the Selection of Sweetened Ultra-Processed Foods and Their Importance in Oral Health. Dent. J..

[38] Assefa D.G., Woldesenbet T.T., Molla W., Zeleke E.D., Simie T.G. (2021). Assessment of knowledge, attitude and practice of mothers/caregivers on infant and young child feeding in Assosa Woreda, Assosa Zone, Benshangul Gumuz Region, Western Ethiopia: A cross-sectional study. Arch. Public Health.

[39] Asante D.O., Dai A., Walker A.N., Zhou Z., Kpogo S.A., Lu R., Huang K., Zou J. (2023). Assessing hypertension and diabetes knowledge, attitudes and practices among residents in Akatsi South District, Ghana using the KAP questionnaire. Front. Public Health.

[40] Dincă F.I., Dimitriu B.A., Săndulescu O., Sîrbu V.D., Săndulescu M. (2024). Knowledge, Attitudes, and Practices of Dental Students from Romania Regarding Self-Perceived Risk and Prevention of Infectious Diseases. Dent. J..

[41] Turki Y., Saleh S., Albaik S., Barham Y., van de Vrie D., Shahin Y., Hababeh M., Armagan M., Seita A. (2020). Assessment of the knowledge, attitudes, and practices (KAP) among UNRWA* health staff in Jordan concerning mental health programme pre-implementation: A cross-sectional study. Int. J. Ment. Health Syst..

[42] Mohammed A.H., Ying L.H., Boon Hong M.L., Sze Nee A.W., Ying L.S., Ramachandram D.S., Hassan B.A. (2024). Development and validation of a knowledge, attitude, and practice (KAP) questionnaire for skin cancer in the general public: KAP-SC-Q. Res. Soc. Adm. Pharm..

[43] UNDP (2021). Knowledge, Attitudes and Perceptions (KAP) Community Survey Towards Women’s Participation in Public Life. https://www.undp.org/sites/g/files/zskgke326/files/migration/jo/00f12b99193a7fe42e9e128a2b06985c527c3c0043f76952c9f763da0159d3eb.pdf.

[44] Majowicz S.E., Hammond D., Dubin J.A., Diplock K.J., Jones-Bitton A., Steven Rebellato S., Leatherdale S.T. (2017). A longitudinal evaluation of food safety knowledge and attitudes among Ontario high school students following a food handler training program. Food Control.

[45] Ma X., Bo L., Zhou X. (2024). Knowledge, attitude, and practice toward foodborne disease among Chinese college students: A cross-sectional survey. Front. Public Health.

[46] O’Brien S.J. (2014). Foodborne Diseases: Prevalence of Foodborne Diseases in Europe. Encycl. Food Saf..

[47] European Food Safety Authority (EFSA) | European Centre for Disease Prevention and Control (ECDC) (2024). The European Union One Health 2023 Zoonoses report. EFSA J..

[48] Tirado C., Schmidt K. (2001). WHO surveillance programme for control of foodborne infections and intoxications: Preliminary results and trends across greater Europe. World Health Organization. J. Infect..

[49] Deac L.M. (2020). Review on Epidemiological Survey on Foodborne Infection in Romania. J. Bacteriol. Infect. Dis..

[50] U.S. Department of Health and Human Services (2017). U.S. Food and Drug Administration. Food Code. https://www.fda.gov/food/resources-you-food/hfp-education-resource-library.

[51] Amodio E., Calamusa G., Tiralongo S., Lombardo F., Genovese D. (2022). A survey to evaluate knowledge, attitudes, and practices associated with the risk of foodborne infection in a sample of Sicilian general population. AIMS Public Health.

[52] Young Y., Waddell L., Harding S., Greig J., Mascarenhas M., Sivaramalingam B., Pham M.T., Papadopoulos A. (2015). A systematic review and meta-analysis of the effectiveness of food safety education interventions for consumers in developed countries. BMC Public Health.

[53] Insfran-Rivarola A., Tlapa D., Limon-Romero J., Baez-Lopez Y., Miranda-Ackerman M., Arredondo-Soto K., Ontiveros S. (2020). A Systematic Review and Meta-Analysis of the Effects of Food Safety and Hygiene Training on Food Handlers. Foods.

[54] European Commission (2005). Commission Regulation (EC) No. 2073/2005 of 15 November 2005 on microbiological criteria for foodstuffs. Off. J. Eur. Union.

[55] Al-Sakkaf A. (2015). Domestic food preparation practices: A review of the reasons for poor home hygiene practices. Health Promot. Int..

[56] Jacob C.J., Powell D.A. (2009). Where does foodborne illness happen-in the home, at foodservice, or elsewhere-and does it matter?. Foodborne Pathog. Dis..

[57] Holst M.M., Wittry B.C., Crisp C., Torres J., Irving D., Nicholas D. (2025). Contributing Factors of Foodborne Illness Outbreaks—National Outbreak Reporting System, United States, 2014–2022. MMWR Surveill. Summ..

[58] Gallo M., Ferrara L., Calogero A., Montesano D., Naviglio D. (2020). Relationships between food and diseases: What to know to ensure food safety. Food Res. Int..

